# Vision loss as the first manifestation of isolated cns relapse in CML blast crisis during flumatinib maintenance therapy: A case report

**DOI:** 10.1097/MD.0000000000050847

**Published:** 2026-09-25

**Authors:** Lili Hong, Jie Mu, Kaiqian Yang, Haifeng Zhuang

**Affiliations:** a Department of hematology, The first People’s Hospital of Hangzhou Linping District, Hangzhou, China; b The First School of Clinical Medicine, Zhejiang Chinese Medical University, Hangzhou, China; c Department of Clinical Hematology and Transfusion, The First Affiliated Hospital of Zhejiang Chinese Medical University (Zhejiang Provincial Hospital of Chinese Medicine), Hangzhou, China.

**Keywords:** chronic myeloid leukemia, diagnostic and therapeutic considerations, Isolated central nervous system relapse, tyrosine kinase inhibitors

## Abstract

**Rationale:**

Central nervous system (CNS) involvement following blast crisis (BC) of chronic myeloid leukemia (CML) represents a challenging complication, particularly when atypical symptoms such as progressive vision loss obscure the diagnosis and delay timely intervention.

**Patient concerns:**

A 65-year-old male presented with isolated CNS relapse following CML-BC, manifesting as progressive visual loss. Despite being in intramedullary complete remission and having received prophylactic intrathecal chemotherapy, the patient’s atypical ocular symptom raised suspicion of CNS involvement.

**Diagnoses:**

Cerebrospinal fluid (CSF) analysis confirmed the presence of blast cells, establishing the diagnosis of CNS relapse.

**Interventions:**

Treatment with intrathecal chemotherapy, systemic high-dose chemotherapy combined with tyrosine kinase inhibitor therapy.

**Outcomes:**

The patient demonstrated visual recovery and sustained disease remission at the 14-month follow-up.

**Lessons:**

This case underscores the importance of clinical vigilance in patients with a history of leukemia who present with blurred vision or progressive vision impairment, as such manifestations,even in the absence of overt neurological symptoms, may be indicators of CNS relapse. Additionally, further investigation through animal models is warranted to characterize the blood-brain barrier penetrance of novel tyrosine kinase inhibitors (TKIs).

## 1. Introduction

Chronic myeloid leukemia (CML) is a clonal myeloproliferative neoplasm characterized by the presence of Philadelphia chromosome, resulting in the BCR::ABL1 fusion gene.^[1]^ The advent of tyrosine kinase inhibitors (TKIs) has revolutionized the treatment of CML, yielding a profound treatment response and prolonged overall survival. With the development of TKIs, survival outcomes have markedly improved.^[2]^ However, once the CML-chronic phase (CP) transforms into CML-BC, the clinical outcomes are very poor.^[3]^ The infiltration of blast cells may involve not only the bone marrow but also extramedullary sites such as the CNS, skin, lymph nodes, soft tissues, and other organs.^[4]^ However, certain TKIs, such as imatinib, exhibit limited blood-brain barrier penetration, which may predispose patients to CNS relapse. Isolated CNS relapse is characterized by blast cell infiltration localized to the CNS, including the brain and spinal cord, without simultaneous involvement of bone marrow and other sites. This condition carries a poor prognosis, with a median survival of less than one year.^[5]^ Despite prophylactic measures during treatment, some patients still experience CNS relapse, which has a significantly adverse impact on treatment outcomes. Isolated CNS relapse following blast crisis in CML poses a distinct clinical challenge. Patients commonly present with neurological symptoms, including dizziness, nausea, and vomiting; nonetheless, visual loss as the initial manifestation is relatively uncommon.^[6]^ This symptom is frequently misdiagnosed as a primary ocular disorder, especially in patients who are in clinical remission, receiving targeted therapy, and not undergoing regular follow-up. However, isolated visual loss may serve as an early warning sign of CNS relapse.

We report a rare case of isolated CNS lymphoblastic crisis relapse occurring during treatment with flumatinib, presenting with progressive visual loss and bilateral optic edema.

## 2. Case presentation

In April 2014, a 65-year-old male was diagnosed with the chronic phase of chronic myeloid leukemia (CML-CP) at an outside hospital and initiated targeted therapy with imatinib 400mg once daily. He achieved a sustained complete cytogenetic remission (CCyR) and major molecular response (MMR). One year later, the patient failed to adhere to medical advice and reduced the imatinib dosage to 200mg daily for maintenance therapy. In December 2020, the patient presented to our hospital with fatigue and fever. Peripheral blood tests revealed a white blood cell (WBC) count of 2.09 × 10^9^/L, a neutrophil count of 0.72 × 10^9^/L, a hemoglobin (Hb) level of 46 g/L, and a platelet (PLT) count of 166 × 10^9^/L. Bone marrow aspirate smears showed 76% blast cells. Flow cytometric analysis indicated that aberrant lymphocytes accounted for 71.48% of cells, with positive expression of CD19, CD10, CD34, TdT, cCD79a, CD123, CD58, cCD22, and CD20, and negative expression of CD117, CD5, CD33, CD13. Karyotypic analysis revealed a karyotype of 50,XY,+X,+del(8)(q22), t(9; 22)(q34; q11.2),+14,add(14)(q32) × 2,der(22)t(9; 22)(q34; q11.2) (20 metaphases analyzed). Quantitative polymerase chain reaction (qPCR) demonstrated a BCR::ABL1 transcript level of 91.96% on the international scale (IS). No ABL1 kinase domain mutation was detected. A final diagnosis of B-cell blast crisis of CML was established.

The patient received induction chemotherapy with the VDCP regimen (vincristine, daunorubicin, cyclophosphamide, prednisone) combined with imatinib 600mg daily for maintenance therapy. He achieved complete hematological remission (CHR), and minimal residual disease (MRD) was negative. After 3 cycles of the combination of high-dose methotrexate and cytarabine, the patient achieved a complete cytogenetic response (CCyR) with a BCR::ABL1 IS level of 0.6%. Additionally, intrathecal chemotherapy (cytarabine, methotrexate, dexamethasone) was administered for the prevention of CNS relapse, and no significant abnormalities were observed in the CSF.

In March 2021, the patient was readmitted for a follow-up examination. Bone marrow examination revealed an MRD level of 0.01% and a BCR::ABL1 transcript level of 52.39% IS. Karyotypic analysis demonstrated the Philadelphia chromosome (translocation between chromosomes 9 and 22), indicating disease progression. The therapeutic regimen was switched to dasatinib 70mg twice daily combined with chemotherapy. Three months later, the patient remained on dasatinib maintenance therapy and attained a major molecular response (MMR), with a P210 transcript level of 0.6%. However, the patient developed dasatinib intolerance, characterized by recurrent pancytopenia, hematuria, edema, and pleural effusion, necessitating a switch to maintenance therapy with flumatinib 400mg daily. Concurrently, intrathecal prophylaxis for CNS relapse was administered at regular intervals. Four months later, follow-up assessments at an outside hospital demonstrated negative results for MRD and achieved MMR. Nevertheless, due to financial constraints, the patient explicitly declined bone marrow transplantation.

On May 5, 2023, the patient was admitted to the hospital with progressive vision loss over several weeks, without dizziness, headache, or other symptoms. Physical examination revealed bilateral limitation in outward eye movement. Ophthalmic examination showed corrected visual acuity of 4.5 in the right eye and 4.8 in the left eye, with an intraocular pressure of 14.7 mm Hg in both eyes, and bilateral lens opacities. The right eye exhibited an optic disc C/D ratio of 0.3, with blurred margins and a visible central foveal reflex in the macular area. Ocular B-scan ultrasound revealed bilateral optic disc edema (Fig. 1). Optical coherence tomography (OCT) of the optic disc demonstrated optic disc edema (Fig. 2). The next day, a follow-up examination showed a corrected visual acuity of 4.2 in the right eye and 4.7 in the left eye, and visual field testing suggested bilateral visual field defects. Orbital magnetic resonance imaging (MRI) (T2-weighted imaging) revealed bilateral optic disc edema, with more severe involvement on the right side (Fig. 3). Cranial MRI revealed multiple areas of pial enhancement. Blood tests showed a WBC count of 5.2*10^9^/L, a neutrophil count of 2.8 × 10^9^/L, an Hb level of 126 g/L, and a PLT count of 183 × 10^9^/L. Bone marrow aspiration revealed no blast cells, chromosomal analysis showed a normal karyotype of 46, XY (20 metaphases analyzed) , with no BCR::ABL1 transcript. Lumbar puncture revealed a CSF pressure of 260 mmH_2_O. CSF flow cytometry showed that immature B lymphocytes accounted for 53.95% of cells, with positive expression of CD19, CD10, CD34, CD123, and TdT, and the BCR::ABL transcript level in the CSF was 0.18%. Based on these findings, a diagnosis of isolated CNS relapse was made. The patient received intrathecal chemotherapy in combination with intravenous chemotherapy (high-dose methotrexate, cytarabine). After seven intrathecal injections, the patient’s vision improved significantly, and CSF test returned to normal. During 14 months of subsequent follow-up, the patient maintained MMR, and MRD of CSF was negative.

**Figure 1.**
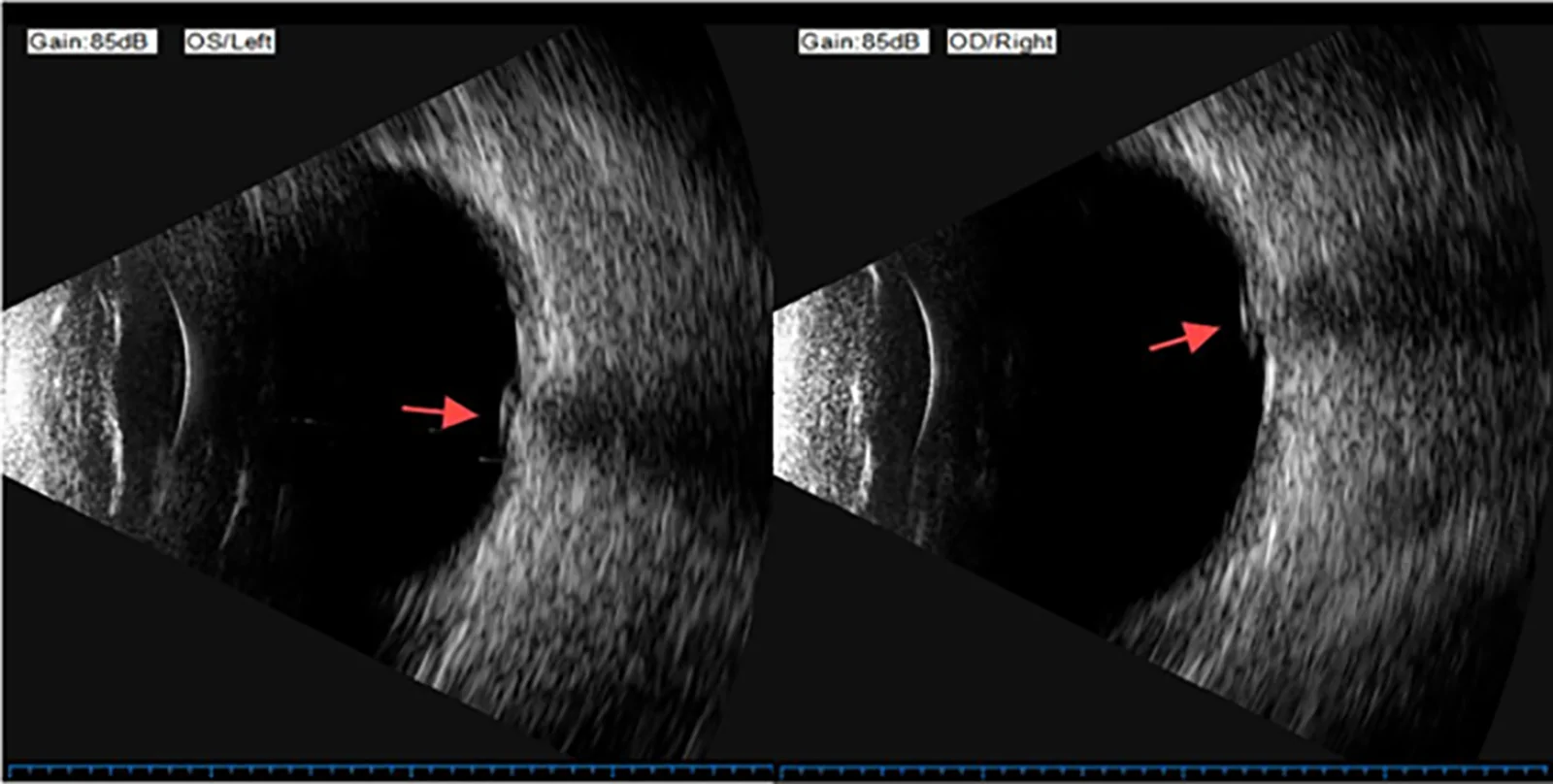
This is an ultrasound image of the patient’s eyes. As indicated by the red arrows in the image, bilateral papilledema is observed.

**Figure 2.**
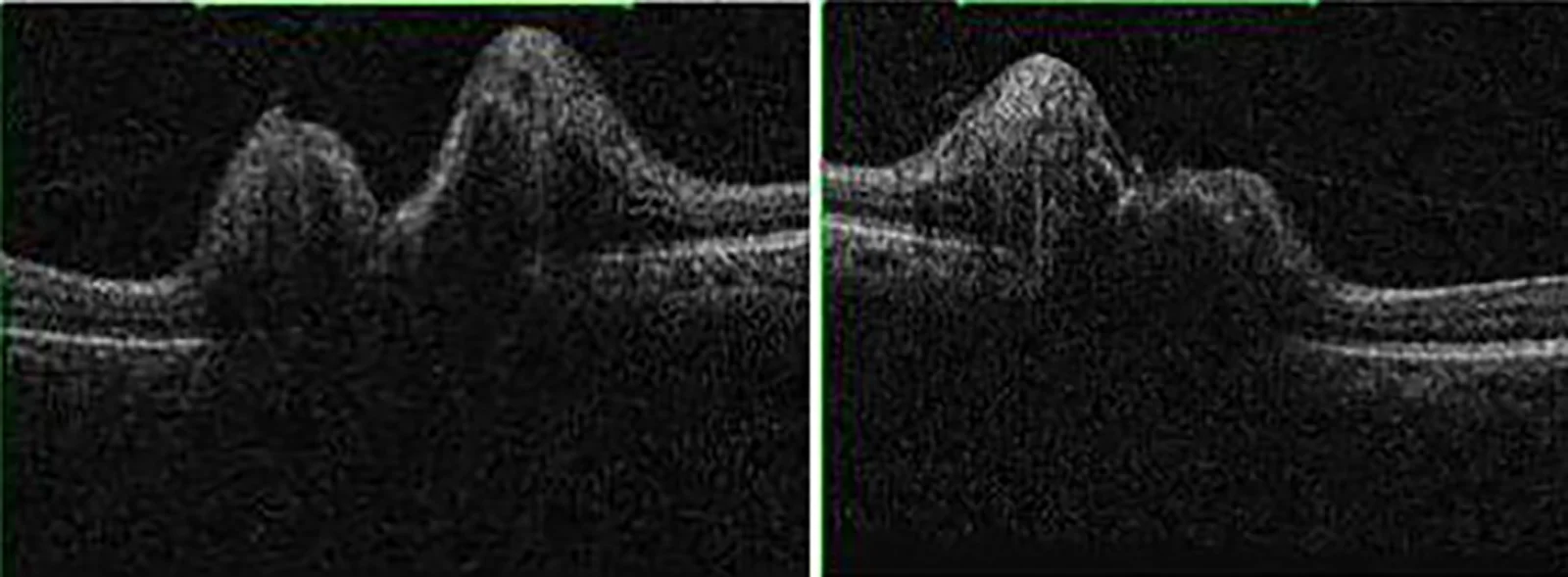
This is an optical coherence tomography (OCT) image of the patient’s optic disc, showing bilateral papilledema.

**Figure 3.**
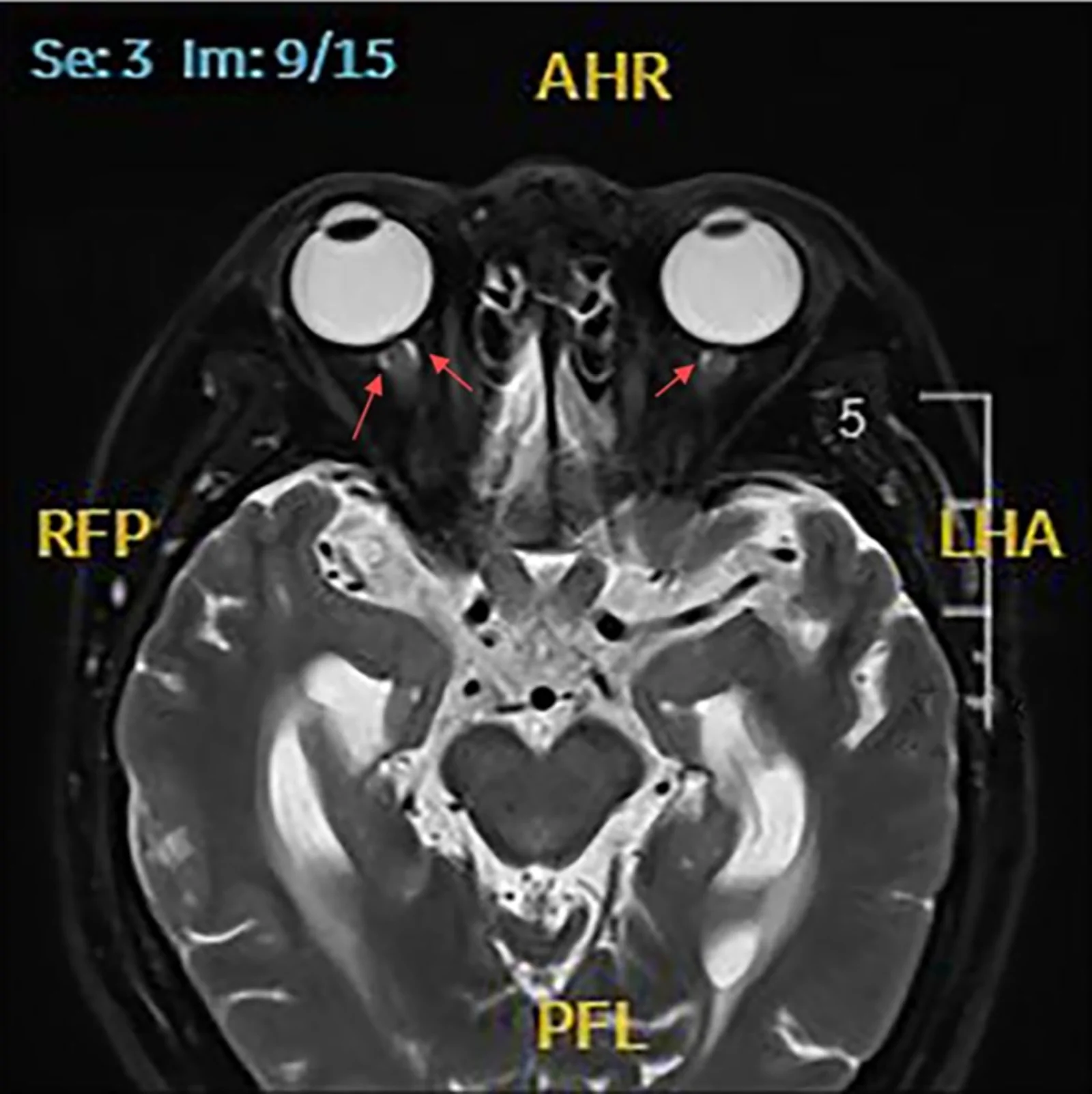
Orbital MRI (T2-weighted imaging) revealed bilateral optic disc edema, with more severe involvement on the right side, as indicated by the red arrows in the images.

## 3. Discussion

We reported a rare case of isolated CNS relapse manifesting as bilateral optic disc edema and progressive visual loss in a CML patient who had previously developed B-cell blast crisis and achieved MMR following treatment.

This case was diagnostically challenging, particularly in the early detection of isolated CNS relapse. Although the classic presentation of CNS relapse includes neurological symptoms such as headache, nausea, vomiting, motor dysfunction, and sensory disturbances, our patient presented with progressive visual loss – a distinctly non-neurological symptom – which is exceptionally rare. Ophthalmic evaluation revealed bilateral optic disc edema as the predominant finding.

The diagnosis was prone to delayed recognition owing to the absence of classic neurological symptoms. In a patient presenting with vision loss and bilateral optic disc edema, the initial differential diagnosis should prioritize the exclusion of primary ocular pathologies, including glaucoma, optic neuritis, and ischemic optic neuropathy. In our patient, preexisting bilateral lens and vitreous opacities may have partially contributed to the baseline visual impairment; however, these findings alone could not account for the acute and progressive visual decline, nor for the associated optic disc edema. Moreover, the constellation of bilateral involvement, the absence of notable intraocular pressure elevation, and the demonstration of pial enhancement on cranial MRI collectively favored the possibility of CNS infiltration. CSF analysis was of critical diagnostic value. In the patient, CSF examination disclosed elevated pressure, a predominance of immature B lymphocytes, and detectable BCR::ABL1 transcripts. Flow cytometry of the CSF demonstrated an immunophenotype (CD19+, CD10+, CD34+, TdT+, cCD79a+, and CD20+) identical to that of the original B-lymphoblastic crisis, providing definitive evidence of clonal identity between the systemic malignancy and the CNS relapse. Therefore, in CML patients who present with unexplained visual impairment – even without overt hematological abnormalities – a high suspicion for CNS relapse is warranted. Definitive diagnosis can be established through magnetic resonance imaging, CSF routine and flow cytometry analysis, and histopathological evaluation. CNS involvement is a rare complication of CML, occurring predominantly in those with blast crisis. Our patient developed CNS relapse insidiously during remission following treatment for lymphoid blast crisis. The protective effect of the blood–brain barrier, which limits the CNS penetration of most TKIs – particularly early-generation agents such as imatinib – is currently considered a key mechanism underlying CNS relapse.^[7]^ Thus, even in the presence of a favorable molecular response in the bone marrow, the CNS may function as a sanctuary site, predisposing patients to isolated CNS relapse.

The management of CNS relapse in CML is particularly challenging due to the limited CNS penetration of many TKIs. Imatinib, the first-line TKI, has poor blood–brain barrier permeability, with CSF concentrations reaching only approximately 5% of plasma levels.^[8]^ Although second-generation TKIs such as dasatinib and nilotinib offer improved CNS penetration, their clinical efficacy in overt CNS involvement remains uncertain.^[9,10]^ Flumatinib, a novel second-generation TKI originating from China, is structurally modified from imatinib through the replacement of the benzene moiety with a pyridine ring and the addition of a trifluoromethyl group.^[11]^ It has demonstrated favorable efficacy and safety profiles in CML patients^[12]^; however, whether it possesses improved blood–brain barrier permeability remains unclear, and data on its CNS penetration are limited. The patient experienced 2 distinct relapses: the first, confined to the bone marrow, was most likely driven by poor treatment adherence; the second, manifesting as isolated CNS disease, involved a more intricate pathogenesis that we postulate is linked to the suboptimal CNS penetrance of TKIs.

To our knowledge, this is the first documented case of isolated CNS blast crisis arising during flumatinib maintenance therapy. Given this finding, appropriate animal models should be established to elucidate the blood–brain barrier permeability of flumatinib, along with its pharmacokinetic behavior and tissue distribution characteristics across a range of dosage regimens. This finding highlights the critical need for routine CNS monitoring in high-risk patients, especially those with a prior history of blast crisis or poor adherence to TKI therapy.

The combination of intrathecal and systemic high-dose chemotherapy represents a well-established therapeutic strategy for CNS relapse. The patient benefited from this approach, with intensive intrathecal therapy and high-dose intravenous methotrexate and cytarabine producing significant symptomatic improvement and CSF normalization. These outcomes are consistent with established guidelines,^[13,14]^ which advocate for early and intensive CNS-directed treatment to minimize the risk of permanent neurological damage. Allogeneic hematopoietic stem cell transplantation (HSCT) remains the only curative modality for CML patients who progress to blast crisis and is recommended for eligible patients achieving a second remission.^[15]^ Although our patient has remained stable over 14 months of follow-up, the aggressive biology of blastic-phase CML, coupled with prior CNS involvement, mandates ongoing close surveillance **–** including regular CSF analysis and ophthalmic evaluation – for early detection of recurrence.

## 4. Conclusion

This case demonstrates that progressive visual loss accompanied by bilateral optic disc edema may serve as an early sign of CNS leukemia relapse. Clinicians should maintain a high suspicion and promptly perform diagnostic evaluations, including CSF analysis, along with timely and aggressive CNS-directed therapy, which are essential for achieving favorable outcomes. Regular CSF and neuroimaging examinations are strongly recommended for early detection of CNS relapse. The management of isolated CNS relapse remains challenging, and further exploration is warranted to investigate TKI pharmacokinetics, blood–brain barrier permeability, the development of novel agents, and optimal dosing strategies for maintenance therapy.

## Acknowledgments

We are grateful to the patient, who gave his informed consent for publication.

## Author contributions

**Data curation:** Lili Hong.

**Resources:** Lili Hong, Haifeng Zhuang.

**Writing – original draft:** Lili Hong, Jie Mu.

**Writing – review & editing:** Lili Hong, Haifeng Zhuang.

**Formal analysis:** Kaiqian Yang.

**Software:** Kaiqian Yang.
